# From engagement to dissemination: a WiseAge patient and public involvement and engagement event on dementia with Lewy bodies

**DOI:** 10.3389/frdem.2026.1919585

**Published:** 2026-09-07

**Authors:** Jon Arild Aakre, Arvid Rongve, Kari Fredriksen, Ellen J. Svendsboe, Ingelin Testad

**Affiliations:** 1 Department of Clinical Medicine, University of Bergen, Bergen, Norway; 2 Centre for Age-Related Medicine - SESAM, Stavanger University Hospital, Stavanger, Norway; 3 Department of Age Related Medicine, Clinic of Medicine, Helse Fonna, Haugesund Hospital, Haugesund, Norway; 4 Department of Health- and Social Sciences, Western Norway University of Applied Sciences, Stord, Norway; 5 Department of Health and Community Sciences, Faculty of Health and Life Sciences, University of Exeter, Exeter, United Kingdom

**Keywords:** ANeED clinical drug trial, dementia research, dementia with Lewy bodies, patient and public involvement and engagement, secondary dissemination, WiseAge

## Abstract

**Background:**

Dementia with Lewy bodies may account for up to 20% of dementia cases, yet public awareness remains limited. Public engagement may help communicate dementia research to wider audiences, but little is known about whether information spreads beyond event attendees. This study explored how attendees evaluated information about dementia with Lewy bodies and the ongoing Ambroxol in New and Early Dementia with Lewy Bodies (ANeED) clinical drug trial presented at a WiseAge public engagement event, and how they reported sharing this information afterwards.

**Methods:**

This descriptive cross-sectional study followed a WiseAge public engagement event that included information about dementia, dementia with Lewy bodies, and the clinical trial. A post-event survey was distributed to attendees, and responses were analysed descriptively.

**Results:**

Of 48 attendees, 18 completed the survey. The information was rated positively, with 16 of 18 rating it as good or very good. Attendees reported sharing information beyond the event, most commonly with friends (*n* = 14) and family members (*n* = 7), while sharing with neighbors was rare (*n* = 1). The number of people with whom information was shared ranged from zero to six. No notable change in trial recruitment was observed during the post-event observation period.

**Conclusion:**

The event was well received and supported secondary dissemination through attendees’ interpersonal networks, particularly friends and family. Although no immediate recruitment effect was observed, public engagement may extend the reach of dementia research communication beyond those who attend.

## 1 Introduction

Dementia with Lewy bodies (DLB) is a neurodegenerative disorder accounting for up to 20% of dementia cases (Aarsland et al., 2008). It is characterized by progressive cognitive impairment, often with fluctuations in attention and alertness, parkinsonian motor features such as tremor, rapid eye movement sleep behavior disorder and a range of neuropsychiatric symptoms, including visual hallucinations and delusions (Mueller et al., 2017; Elder et al., 2022). Compared with Alzheimer’s disease (AD), DLB is often associated with greater clinical complexity, higher caregiver burden, and poorer overall outcomes (Svendsboe et al., 2016; Mueller et al., 2017). Despite being the second most common form of neurodegenerative dementia, awareness of the condition among the general public, as well as among health workers, general practitioners and even hospital specialists remains limited (Agarwal et al., 2024). Consequently, patients may end up seeing multiple clinical specialists before a correct diagnosis is made, be misdiagnosed with AD or Parkinson’s disease, or remain undiagnosed. Patients and caregivers are thus emphasizing the need for improved education and awareness of DLB both to the public and healthcare systems and professionals (Armstrong et al., 2020), and increasing public awareness is also recognized as an essential strategy for reducing dementia-related stigma (Hung et al., 2021).

Patient and public involvement and engagement (PPIE) in research is an overarching term describing activities through which patients and the public contribute to, or engage with, research. PPIE has the potential to improve the quality and relevance of dementia research (Gove et al., 2018). However, involvement and engagement remain conceptually unclear in the literature, with multiple and sometimes overlapping definitions (Wilkinson et al., 2024). Patient and public involvement (PPI), drawing on the definition developed by INVOLVE, refers to research being carried out “with” or “by” members of the public rather than “to,” “about” or “for” them, emphasizing active contribution and partnership in the research process, with training and support identified as important for meaningful involvement (Staley, 2009). In the context of this study, patients refer to individuals living with dementia with Lewy bodies, while the public may include people at risk of dementia, members of the general public, informal carers, and individuals who use, or have used, dementia-related health and social care services (Georges et al., 2022) as well as relevant user organizations and advocacy groups. Public engagement has its origins in science communication, where earlier approaches were often characterized by a “deficit model” focused on improving public understanding of science, but has increasingly evolved toward more dialog-based and participatory processes (Wilkinson et al., 2024). Within this context, public and patient engagement (PPE) refers to activities aimed at increasing awareness of research and recruitment, encouraging interest in research topics, and communicating knowledge and findings to patients and the broader public.

In Norway, recruitment to dementia clinical studies, including academic drug trials, is largely embedded within regional healthcare systems, with participant identification and inclusion typically occurring through regular clinical services. However, only one third of individuals with dementia receive a formal diagnosis, often during the later disease stages (Gjøra et al., 2024). PPI in the design of clinical trials may improve participant recruitment (Crocker et al., 2018), while PPE activities may complement clinical recruitment pathways by increasing awareness of the disease and of research opportunities among individuals not yet engaged with healthcare services (Langbaum et al., 2023).

WiseAge is the Centre for Age-Related Medicine’s (SESAM) organizational platform for user involvement and societal engagement that ensures active user participation in all research at the centre, with the aim of enhancing research relevance and usefulness. Members are aged sixty or older, with a wide variety of backgrounds and knowledge. User representatives from WiseAge collaborate with researchers at SESAM across all phases of new and ongoing research projects, including dissemination. WiseAge organizes both virtual and in-person events to engage the public and its members in research, supporting knowledge exchange, dissemination of information about ageing-related conditions and ongoing research studies, thus providing a structured approach to PPE.

This study aimed to explore how information presented in the main program element, a one-hour lecture on DLB at a general WiseAge PPE event, was perceived and disseminated. The lecture focused on dementia, dementia with Lewy bodies, and the ongoing clinical study Ambroxol in New and Early Dementia with Lewy Bodies (ANeED) (Chwiszczuk et al., 2023), an 18-month randomized controlled trial followed by a 12-month open-label extension, with a parallel study exploring caregiver distress and collecting digital sensor data to develop digital biomarkers.

## 2 Method

### 2.1 Study design

This study used a descriptive design with a cross-sectional survey administered following a public engagement event. PPIE in this study is reported in accordance with the Guidance for Reporting Involvement of Patients and the Public 2 (GRIPP 2)—short form checklist (Staniszewska et al., 2017), please see Supplementary material 1—GRIPP 2 reporting checklist. Co-author Kari Fredriksen (KF), leader of the WiseAge user panel, was involved in this study as the user representative. PPIE in this study aimed to ensure that the study design, interpretation of findings, and reflections on public engagement activities were informed by user perspectives throughout the process.

### 2.2 Ethical considerations

The study was conducted in accordance with the General guidelines for research ethics of the Norwegian National Research Ethics Committees (Norwegian National Committees for Research Ethics, 2019). Approval from the Regional Committees for Medical and Health Research Ethics (REK) was not required, as the study did not constitute medical and health research under the Norwegian Health Research Act. Participation was voluntary, survey responses were collected anonymously, and completion of the survey was considered consent to participate.

### 2.3 WiseAge PPIE infrastructure

**Figure 1:**
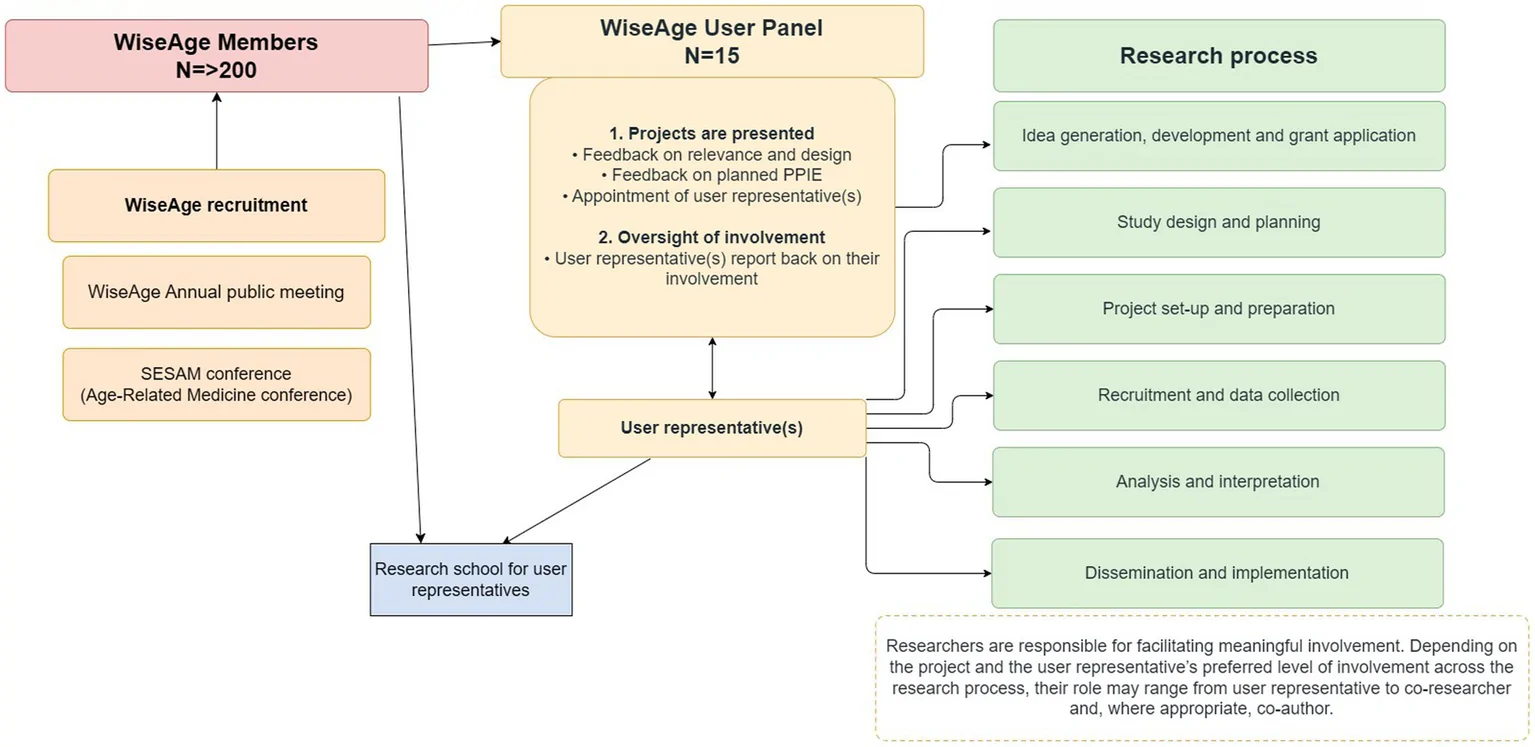
Overview of the WiseAge PPIE structure and involvement across the research process. The figure illustrates the relationship between WiseAge members, the User Panel, appointed user representatives, and their involvement across different phases of research. It does not represent the extent of influence exercised at each stage in the present study.

WiseAge, established in 2015 by SESAM for user involvement and societal engagement, provides the organizational context for the public engagement event examined in this study and, by May 2026, had 209 members. Central to this structure is the WiseAge User Panel, an advisory group of up to 15 WiseAge members with diverse life experiences and knowledge. This group has regular meetings to provide feedback to research projects on study relevance, design, and planned PPIE activities and is responsible for assigning user representatives to the projects. The appointed user representatives follow the study throughout its duration to ensure PPIE across all phases of the research process. The User Panel also monitors the progress of ongoing projects and helps ensure PPIE remains active through the research process. In addition to project-specific involvement, WiseAge and SESAM support broader PPIE activities through research schools, collaborative forums, and public events that facilitate interaction, knowledge exchange, and engagement between researchers, user representatives, and the wider public (Figure 1).

### 2.4 Public meeting

The WiseAge annual public meeting was held at a hotel venue on 26 October 2023 in Stavanger, Norway. The event was open to the public and free of charge and included lunch. Invitations to the meeting were distributed via email to WiseAge members (*n* = 103) and through an open announcement on the WiseAge Facebook page (1,504 followers at the time of distribution). The meeting was advertised as an event for people aged 60 years or older. A total of 48 individuals attended the event.

The program included an introduction to WiseAge and an update on SESAM research activities, followed by the main program element, a one-hour in-person lecture delivered by two researchers: a professor and geriatric psychiatrist and a postdoc and geriatrician from the ANeED study. The lecture covered dementia in general, dementia with Lewy bodies, and the ANeED study with visual support information provided via powerpoint. The event also included a cultural segment.

### 2.5 Survey

An initial 12-question draft was developed and refined by researchers from the ANeED study team, including a postdoctoral researcher, the study’s principal investigator, Arvid Rongve (AR), and Ingelin Testad (IT), a work package lead in the ANeED study and director of SESAM. The draft was further revised during an in-person working session at SESAM involving KF, IT, and another SESAM staff member. During this process, the questionnaire was reduced from 12 to six questions, and an optional open-ended field for additional comments was added. The survey setup in Corporater Surveyor was tested before distribution to verify correct functionality and data capture; however, the questionnaire was not pilot-tested with members of the target population. The final questionnaire is provided in Supplementary material 2—Survey.

The questionnaire assessed attendees’ perceptions of the information presented at the PPE event, its subsequent sharing, familiarity with dementia with Lewy bodies, knowledge of anyone who might wish to participate in the ANeED study, and exposure to related social media content. Respondents rated the information about dementia with Lewy bodies as “very good,” “good,” or “less good”; reported the number of people with whom they had shared it by entering a number; and could select multiple recipient categories (family, friends, and neighbors). The remaining three questions used yes/no response options and asked whether respondents knew someone with dementia with Lewy bodies, knew someone who might wish to participate in the ANeED study, and had seen SESAM’s Facebook post on WiseAge containing clips from the event. An optional open-ended field allowed respondents to provide additional comments.

### 2.6 Analysis

Data were analysed descriptively using frequencies and counts for categorical variables. Given the exploratory nature of the study and the small sample size, no inferential statistical analyses were performed.

## 3 Results

### 3.1 Survey responses

Eighteen attendees completed the survey. None of the respondents reported knowing someone with DLB, knowing someone interested in participating in the ANeED study, or having been exposed to post-event content on social media.

**Figure 2:**
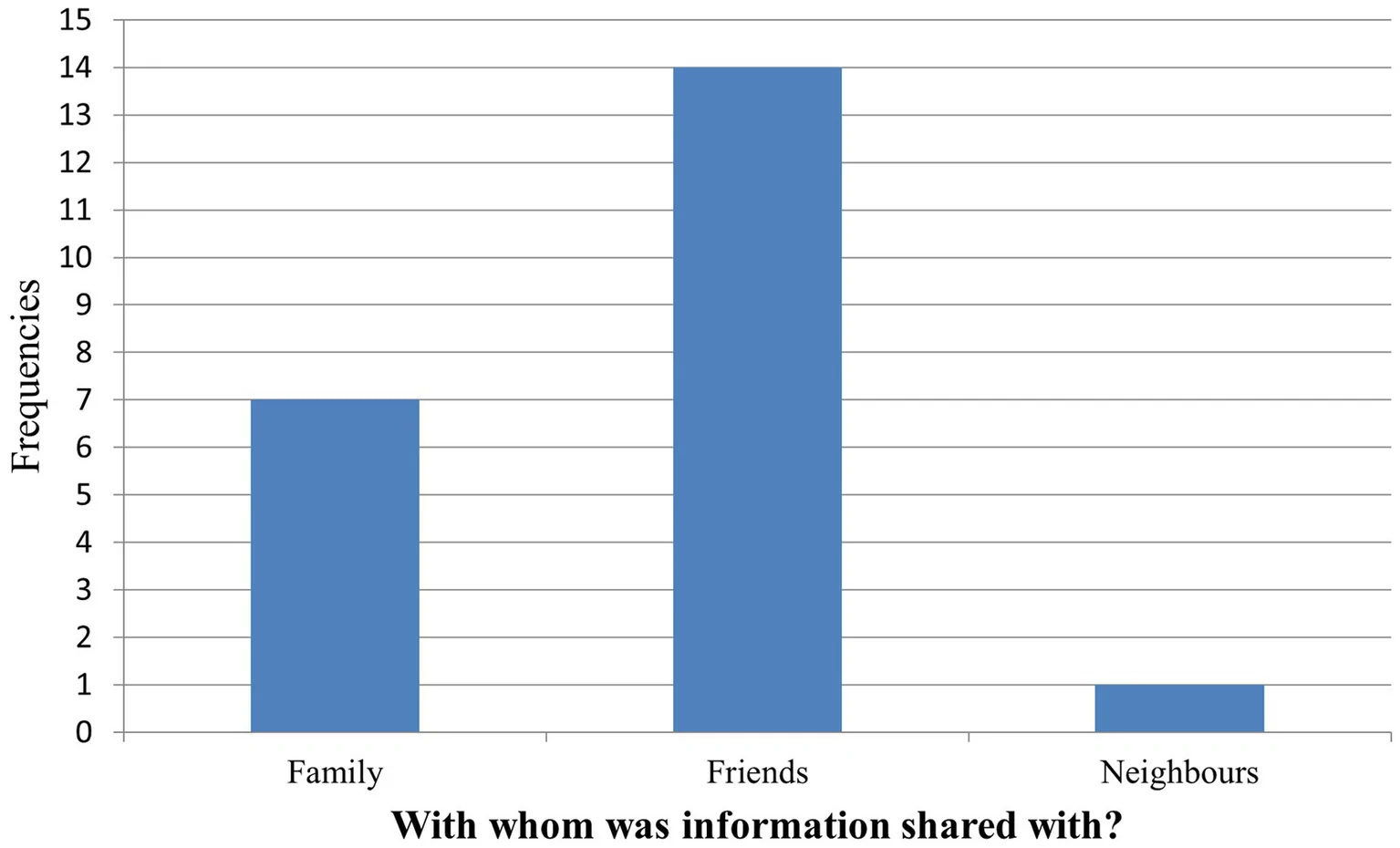
Categories of individuals with whom attendees shared information.

As Figure 2 shows, information was mostly shared with friends (*n* = 14) and family members (*n* = 7), while few attendees reported sharing information with neighbors (*n* = 1).

**Figure 3:**
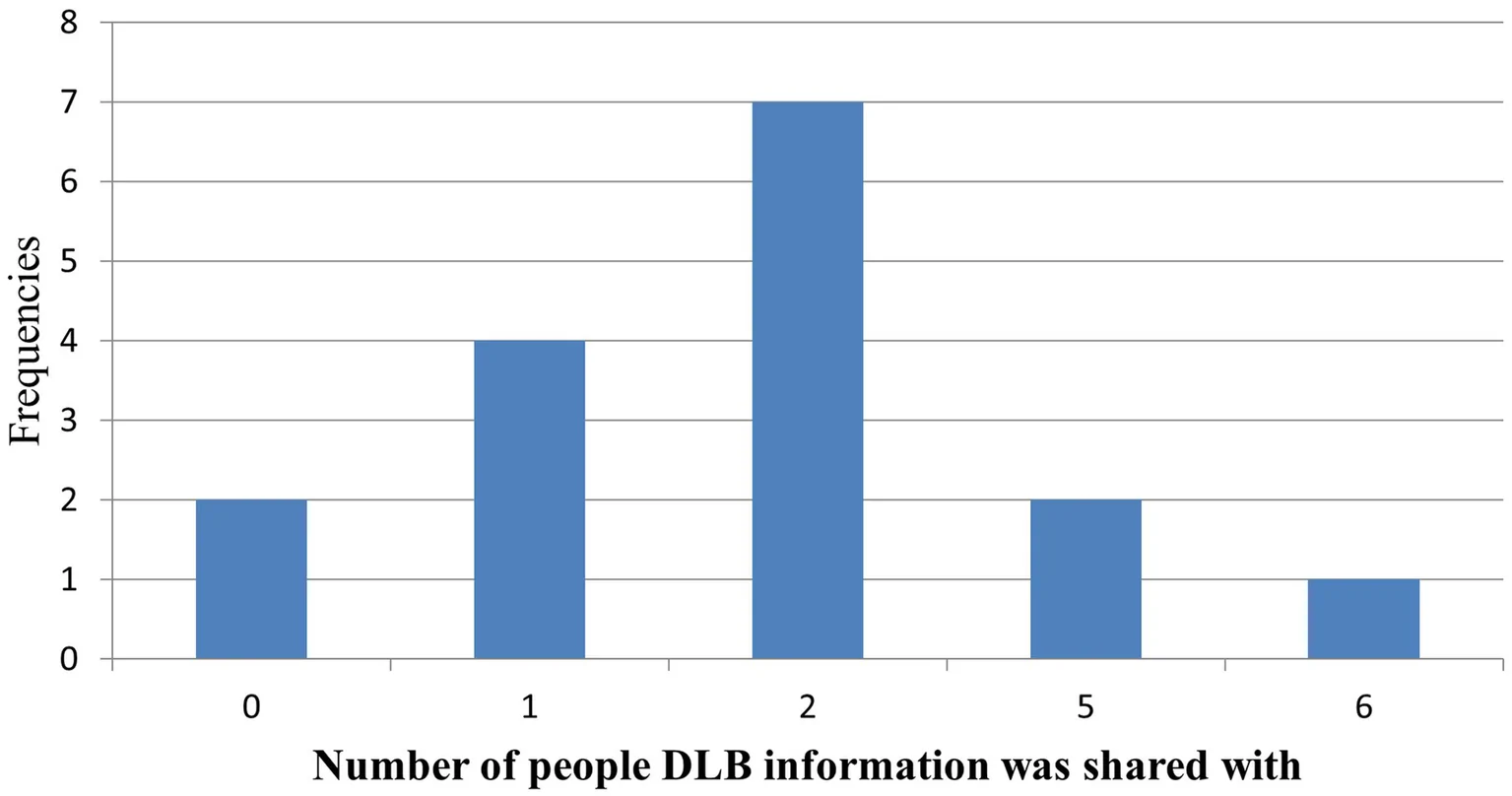
Number of individuals with whom attendees reported sharing information.

Among the 16 respondents who answered this question, the reported number of people with whom information was shared ranged from zero to six (Figure 3).

**Figure 4:**
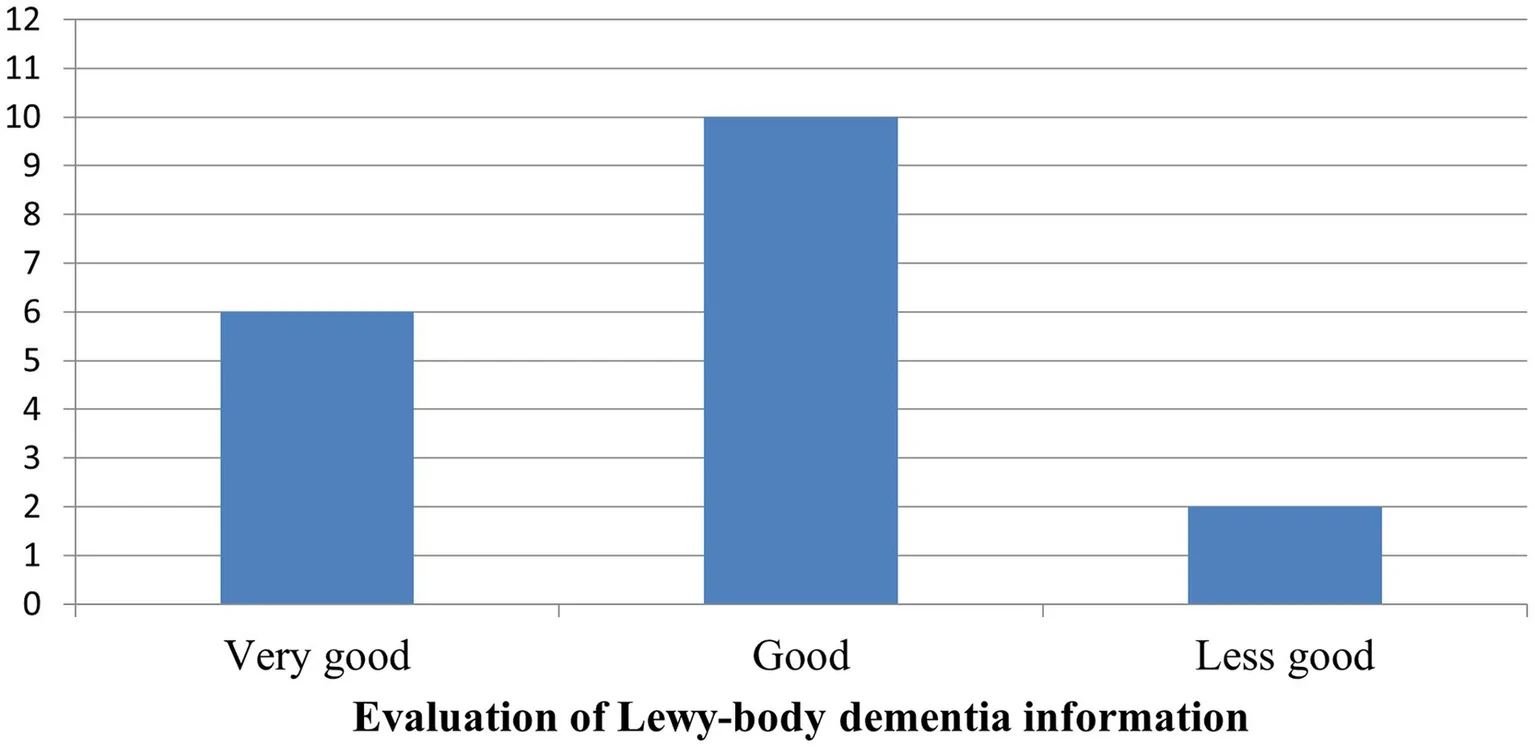
Evaluation of information about dementia with Lewy bodies among attendees.

As Figure 4 shows, most respondents evaluated the information about dementia with Lewy bodies positively. Ten attendees rated the information as good and six as very good, while two rated it as less good.

### 3.2 Recruitment

No notable change in ANeED study recruitment was observed between the public meeting on 26 October 2023 and the distribution of the follow-up questionnaire on 8 December 2023.

## 4 Discussion

This study evaluated selected aspects of a public engagement event related to the ANeED clinical study, focusing on attendees’ perceptions of the information presented and the secondary dissemination of this information beyond the event. A key contribution of this study is the provision of insights into how and with whom information about dementia with Lewy bodies and the DLB trial ANeED is shared beyond direct attendees, offering insight into the downstream effects of secondary dissemination following public engagement activities.

One of the most substantial barriers to recruitment in dementia clinical studies is that potential participants are never reached or made aware of research opportunities (Langbaum et al., 2023). However, no notable change in recruitment rates to the ANeED clinical study was observed during the short period between the public event and post event survey. This is consistent with survey findings indicating that none of the respondents reported knowing someone with dementia with Lewy bodies or someone who might be interested in participating in the study, suggesting that the event had limited immediate relevance for direct recruitment. Despite this, increasing awareness and engagement around DLB through public engagement activities represent an important initial step and may foster recruitment in the long run, potentially facilitated by the information sharing with friends and family.

The reported sharing of information with others following the event is a key finding of this study, providing an indication that information was shared beyond event attendees through their social networks. Information was most commonly shared with friends and family members, while sharing with neighbors was rare. Similar patterns have also been identified in the broader health care service literature, suggesting that knowledge may be sought and shared across social networks and communities, including through word-of-mouth (Bhowmick et al., 2025). This form of secondary dissemination is rarely captured in evaluations of public engagement activities and may represent an important but underexplored pathway for increasing awareness of DLB and clinical study opportunities in the community, as such informal exchanges may shape health-related decisions (Bhowmick et al., 2025).

Meeting attendees evaluated the information about dementia with Lewy bodies positively, with the majority rating it as good or very good. This suggests that the one-hour presentation was perceived as accessible and acceptable at this public event, an important condition for effectively increasing awareness in this context, however, knowledge acquisition or retention was not assessed, and the extent to which attendees gained new insight remains unclear.

From a practical perspective, findings from our study suggest that public engagement activities may facilitate dissemination through attendees’ interpersonal networks, and that this downstream information sharing should be explicitly taken into account when planning how information is communicated and distributed. Greater emphasis could be placed on equipping attendees with clear, concise, and accessible materials specifically for secondary audiences, such as brief written summaries or handouts that can be easily shared with others. Such materials could be co-produced with diverse PPIE groups, including people affected by dementia, to adapt language, format, and communication approaches to specific audiences (de Wolf-Linder et al., 2024). In the context of dementia with Lewy bodies, where awareness of the condition remains limited, co-production workshops may help improve the relevance and accessibility of materials intended to raise awareness. Printed materials could also include trackable QR codes linking to reliable digital resources, providing objective indicators of reach, such as the number of QR-code scans or page visits. Such indicators, combined with prospective follow-up of attendees, could support assessment of how information moves through social networks.

The structured and continuous nature of these yearly public engagement events, jointly planned by WiseAge and researchers, may facilitate the involvement of project-specific user representatives in the development, presentation of and dissemination of study materials. Such involvement may support their inclusion and active participation in the study, which is a prerequisite for meaningful PPIE (Staley, 2009). This approach may also help ensure that communication strategies are more closely aligned with the needs and perspectives of intended audiences.

This study has several limitations. The sample size was small, data were based on self-report, and participation in the survey was voluntary, introducing the possibility of selection bias. Recipients of the shared information were not assessed directly, limiting assessment of the actual secondary dissemination reach. The questionnaire did not collect sociodemographic characteristics, including age and gender, WiseAge membership status, professional background, caregiving experience, whether respondents were themselves living with dementia, including DLB, or their prior familiarity with dementia—thereby limiting assessment of the respondent groups and social networks represented, the representativeness of the sample, and the generalisability of the findings.

The established working relationship with KF, as leader of the WiseAge user panel, facilitated her early involvement in both questionnaire development and manuscript preparation. During the in-person working session, KF contributed to the revision of the initial researcher-developed questionnaire, including the decision to reduce it from 12 to six questions and include an optional open-ended comment field. After the first author prepared the initial manuscript draft, KF participated in discussions of the findings and their interpretation. Her input led to the inclusion of practice-oriented reflections on how future WiseAge PPE events, or similar initiatives, could be arranged; these reflections had not been included in the initial draft. Proposed questionnaire and manuscript revisions were discussed and agreed jointly before finalization, and no notable disagreements arose during these discussions. KF also critically reviewed and approved the final manuscript as a co-author.

The study provides insights into how public engagement activities may function within a PPIE context, particularly in facilitating secondary dissemination through interpersonal networks. These findings also highlight the importance of considering social networks of attendees when planning and distributing study materials in public engagement events. Future studies should prospectively examine secondary dissemination through attendees’ social networks over time, potentially using digital tools to provide objective indicators of its reach.

## Data Availability

The raw data supporting the conclusions of this article will be made available by the authors, without undue reservation.

## References

[ref1] Aarsland D. Rongve A. Piepenstock Nore S. Skogseth R. Skulstad S. Ehrt U. . (2008). Frequency and case identification of dementia with Lewy bodies using the revised consensus criteria. Dement. Geriatr. Cogn. Disord. 26, 445–452. doi: 10.1159/000165917, 18974647

[ref2] Agarwal K. Backler W. Bayram E. Bloom L. Boeve B. F. Cha J. H. . (2024). Lewy body dementia: overcoming barriers and identifying solutions. Alzheimers Dement. 20, 2298–2308. doi: 10.1002/alz.13674, 38265159 PMC10942666

[ref3] Armstrong M. J. Gamez N. Alliance S. Majid T. Taylor A. Kurasz A. M. . (2020). Research priorities of caregivers and individuals with dementia with Lewy bodies: an interview study. PLoS One 15:e0239279. doi: 10.1371/journal.pone.0239279, 33027276 PMC7540843

[ref4] Bhowmick A. van der Zande M. M. Harris R. (2025). Knowledge-seeking and knowledge sharing of health services across social networks and communities: a scoping review. BMC Health Serv. Res. 25:443. doi: 10.1186/s12913-025-12525-y, 40148964 PMC11948718

[ref5] Chwiszczuk L. J. Breitve M. H. Kirsebom B. B. Selnes P. Fløvig J. C. Knapskog A. B. . (2023). The ANeED study—ambroxol in new and early dementia with Lewy bodies (DLB): protocol for a phase IIa multicentre, randomised, double-blinded and placebo-controlled trial. Front. Aging Neurosci. 15:1163184. doi: 10.3389/fnagi.2023.1163184, 37304077 PMC10250712

[ref6] Crocker J. C. Ricci-Cabello I. Parker A. Hirst J. A. Chant A. Petit-Zeman S. . (2018). Impact of patient and public involvement on enrolment and retention in clinical trials: systematic review and meta-analysis. BMJ 363:k4738. doi: 10.1136/bmj.k4738, 30487232 PMC6259046

[ref7] de Wolf-Linder S. Kramer I. Hersperger M. Schubert M. Bächi S. Stolz M. . (2024). Meaningful patient and public engagement in dissemination-embedding co-production in dementia research. Front. Dement. 3:1426019. doi: 10.3389/frdem.2024.1426019, 39351041 PMC11439697

[ref8] Elder G. J. Lazar A. S. Alfonso-Miller P. Taylor J. P. (2022). Sleep disturbances in Lewy body dementia: a systematic review. Int. J. Geriatr. Psychiatry 37:e5814. doi: 10.1002/gps.5814, 36168299 PMC9827922

[ref9] Georges J. Diaz-Ponce A. Lamirel D. Moradi-Bachiller S. Gove D. (2022). Keeping track of and recognizing the value of public involvement work in dementia research. Front. Neurol. 13:1031831. doi: 10.3389/fneur.2022.1031831, 36438974 PMC9691954

[ref10] Gjøra L. Strand B. H. Bergh S. Bosnes I. Johannessen A. Livingston G. . (2024). Prevalence and determinants of diagnosed dementia: a registry linkage study linking diagnosis of dementia in the population-based HUNT study to registry diagnosis of dementia in primary care and hospitals in Norway. J. Alzheimer's Dis 99, 363–375. doi: 10.3233/jad-240037, 38701153 PMC11091616

[ref11] Gove D. Diaz-Ponce A. Georges J. Moniz-Cook E. Mountain G. Chattat R. . (2018). Alzheimer Europe's position on involving people with dementia in research through PPI (patient and public involvement). Aging Ment. Health 22, 723–729. doi: 10.1080/13607863.2017.1317334, 28513210

[ref12] Hung L. Hudson A. Gregorio M. Jackson L. Mann J. Horne N. . (2021). Creating dementia-friendly communities for social inclusion: a scoping review. Gerontol. Geriatr. Med. 7:23337214211013596. doi: 10.1177/23337214211013596, 34036118 PMC8127744

[ref13] Langbaum J. B. Zissimopoulos J. Au R. Bose N. Edgar C. J. Ehrenberg E. . (2023). Recommendations to address key recruitment challenges of Alzheimer's disease clinical trials. Alzheimers Dement. 19, 696–707. doi: 10.1002/alz.12737, 35946590 PMC9911558

[ref14] Mueller C. Ballard C. Corbett A. Aarsland D. (2017). The prognosis of dementia with Lewy bodies. Lancet Neurol. 16, 390–398. doi: 10.1016/s1474-4422(17)30074-1, 28342649

[ref15] Norwegian National Committees for Research Ethics. (2019) *General guidelines for research ethics* [Online]. Oslo: Norwegian National Committees for Research Ethics. Available online at: https://www.forskningsetikk.no/en/guidelines/general-guidelines/ (Accessed June 2, 2026).

[ref16] Staley K. (2009). Exploring impact: Public Involvement in NHS, public Health and social care Research. Eastleigh: INVOLVE.

[ref17] Staniszewska S. Brett J. Simera I. Seers K. Mockford C. Goodlad S. . (2017). GRIPP2 reporting checklists: tools to improve reporting of patient and public involvement in research. Res. Involv. Engagem. 3:13. doi: 10.1186/s40900-017-0062-2, 29062538 PMC5611595

[ref18] Svendsboe E. Terum T. Testad I. Aarsland D. Ulstein I. Corbett A. . (2016). Caregiver burden in family carers of people with dementia with Lewy bodies and Alzheimer's disease. Int. J. Geriatr. Psychiatry 31, 1075–1083. doi: 10.1002/gps.4433, 26765199

[ref19] Wilkinson C. Gibson A. Biddle M. Hobbs L. (2024). Public involvement and public engagement: an example of convergent evolution? Findings from a conceptual qualitative review of patient and public involvement, and public engagement, in health and scientific research. PEC Innov. 4:100281. doi: 10.1016/j.pecinn.2024.100281, 38638421 PMC11024997

