# Supplementary material for: From engagement to dissemination: a WiseAge patient and public involvement and engagement event on dementia with Lewy bodies
Source: Front Dement. 2026 Sep 7;5:1919585. doi: 10.3389/frdem.2026.1919585 (PMC13590376; doi:10.3389/frdem.2026.1919585)
Supplement: Supplementary file 1 [file Data_Sheet_1.docx]

| **Section and topic** | **Item** | **Reported on page No.** |
| --- | --- | --- |
| **1: Aim** | Report the aim of PPI in the study | p. 2 |
| **2: Methods** | Provide a clear description of the methods used for PPI in the study | pp. 2-3 |
| **3: Study results** | Outcomes—Report the results of PPI in the study, including both positive and negative outcomes | p. 6 |
| **4: Discussion and conclusions** | Outcomes—Comment on the extent to which PPI influenced the study overall. Describe positive and negative effects | pp. 6 |
| **5: Reflections/critical perspective** | Comment critically on the study, reflecting on the things that went well and those that did not, so others can learn from this experience | p. 6 |

Staniszewska S, Brett J, Simera I, Seers K, Mockford C, Goodlad S, Altman DG, Moher D, Barber R, Denegri S, Entwistle A, Littlejohns P, Morris C, Suleman R, Thomas V, Tysall C. GRIPP2 reporting checklists: tools to improve reporting of patient and public involvement in research. Res Involv Engagem. 2017 Aug 2;3:13. doi: 10.1186/s40900-017-0062-2. PMID: 29062538; PMCID: PMC5611595.
