# Supplementary material for: From engagement to dissemination: a WiseAge patient and public involvement and engagement event on dementia with Lewy bodies
Source: Front Dement. 2026 Sep 7;5:1919585. doi: 10.3389/frdem.2026.1919585 (PMC13590376; doi:10.3389/frdem.2026.1919585)
Supplement: Supplementary file 2 [file Data_Sheet_2.DOCX]

Attachment 1: Survey (translated from Norwegian to English)

**ANeED is a clinical study of Ambroxol in early-stage dementia with Lewy bodies.**

Dementia with Lewy bodies (DLB) is the second most common form of dementia.

DLB causes characteristic symptoms such as cognitive impairment, visual hallucinations, sleep problems, and Parkinson-like features such as tremors and slowed movements.

DLB is a type of dementia that is often underdiagnosed and misdiagnosed.

There has also been very little research on it. There is no treatment that can slow the progression of the disease or cure it.

Through the ANeED study, we hope to come one step closer to such a treatment.

We would like to ask you a few questions about this topic and the presentation given by Miguel Borda and Arvid Rongve during the WiseAge public meeting. It will take approximately 5 minutes to answer all the questions.

Thank you for participating in this survey!

1. **How was the information about dementia with Lewy bodies?**
   [ ] Very good
   [ ] Good
   [ ] Less good
2. **How many people have you shared this information with?**

[Enter number]

3. **Who did you share the information with? You may select more than one answer.**
   [ ] Family
   [ ] Friends
   [ ] Neighbours
4. **Do you know anyone who has dementia with Lewy bodies?**
   [ ] Yes
   [ ] No
5. **Do you know anyone who would like to participate in the ANeED research project?**
   [ ] Yes
   [ ] No
6. **Did you see the Facebook post on WiseAge by SESAM, with clips from that day?**
   [ ] Yes
   [ ] No

Thank you for participating in this survey! If you have any additional comments, you may write them below:
